# Integrated Karyotypes of Diploid and Tetraploid Carrizo Citrange (*Citrus sinensis* L. Osbeck × *Poncirus trifoliata* L. Raf.) as Determined by Sequential Multicolor Fluorescence *in situ* Hybridization With Tandemly Repeated DNA Sequences

**DOI:** 10.3389/fpls.2020.00569

**Published:** 2020-05-27

**Authors:** Honghong Deng, Guohao Tang, Nuo Xu, Zhijian Gao, Lijin Lin, Dong Liang, Hui Xia, Qunxian Deng, Jin Wang, Zexi Cai, Guolu Liang, Xiulan Lv

**Affiliations:** ^1^Institute of Pomology and Olericulture, Sichuan Agricultural University, Chengdu, China; ^2^College of Horticulture and Landscape Architecture, Southwest University, Chongqing, China; ^3^National Maize Improvement Center, College of Agronomy and Biotechnology, China Agricultural University, Beijing, China

**Keywords:** citrus rootstock, satellite repeat, ribosomal DNA, oligonucleotide probe, cytogenetic markers, chromosome discrimination

## Abstract

Carrizo citrange [*Citrus sinensis* (L.) Osbeck × *Poncirus trifoliata* (L.) Raf., CC] is one of the most widely used rootstocks in citriculture worldwide, but its cytogenetic study has been hampered by its inherent small size, morphological similarity to mitotic chromosomes, and lack of accessible cytological landmarks. In our previous study, a spontaneously occurring tetraploid CC seedling was discovered. The main goals of this study were to elucidate the chromosome constitution and construct the karyotypes of diploid CC rootstock and its corresponding spontaneously occurring tetraploid. To accomplish these, the chromosomal characteristics were investigated by sequential multicolor fluorescence *in situ* hybridization (FISH) with eight properly labeled repetitive DNA sequences, including a centromere-like repeat, four satellite repeats, two rDNAs, and an oligonucleotide of telomeric (TTTAGGG)_*n*_ repeat. The results nicely demonstrated that these repetitive DNAs are reliable cytogenetic markers that collectively facilitate simultaneous and unequivocal identification of homologous chromosome pairs. Based on chromosome size and morphology together with FISH patterns of repetitive DNAs, an integrated karyotype of CC rootstock was constructed, consisting of 2n = 2x = 12m (1sat) + 6sm with karyotype asymmetry degree being divided into 2B category. Cytogenetically speaking, the variable and asymmetric distribution patterns of these repetitive DNAs were fully confirmed the hybrid nature of CC rootstock. In addition, comparative distribution patterns and chromosomal localizations of these repetitive DNAs convincingly showed that this tetraploid CC material arose from somatic chromosome doubling of diploid CC rootstock. This study revealed, for the first time, the integrated karyotype and chromosomal characteristics of this important citrus rootstock as well as its spontaneously occurring tetraploid plant. Furthermore, this study is a good prospective model for study species with morphologically indistinguishable small chromosomes.

## Introduction

Citrus is one of the most horticulturally and economically important fruit crops globally (Wu et al., 2018), extensively cultivated in more than 140 countries and regions in the tropics and subtropics (Cuenca et al., 2018). Citrus cultivars are grafted on the rootstock for commercial production worldwide (Oustric et al., 2017, 2019; Bowman and Joubert, 2020). Citrus scion cultivars may respond differently to vegetative growth, canopy size, fruit quality and production, resistance to pests and diseases, as well as tolerance to various abiotic and biotic stresses when grown on diverse rootstocks (Warschefsky et al., 2016; Wang et al., 2017; Oustric et al., 2019; Bowman and Joubert, 2020). Thus, the use of proper rootstock is utmost importance to the success in growing attractive and productive citrus fruits (Albrecht et al., 2019; Bowman and Joubert, 2020).

Carrizo citrange [*Citrus sinensis* (L.) Osbeck × *Poncirus trifoliata* (L.) Raf., CC] is a generic hybrid (Deng, 2008) of a Washington navel orange and a trifoliate orange that was obtained almost a century ago (Huerta et al., 2009). Currently, CC remains one of the most commonly used rootstocks in citriculture worldwide, especially in China (Deng, 2008), Spain (Ruiz et al., 2016b), the United States (Albrecht et al., 2012), and several other major citrus-producing countries. The main advantages of CC are excellent tolerance to disease and flooding, intermediate resistance to *Phytophthora* root rot, great contribution to fruit quality and yield, and good compatibility with citrus scion cultivars (Bowman and Joubert, 2020). For instance, one of the most important strategies for controlling citrus tristeza virus (CTV) employs the utilization of tolerant CC rootstock (Castle et al., 2009). Additionally, CC rootstock has been described as having good cold hardiness for citrus scion cultivars (Oustric et al., 2017). The multibillion-dollar citrus industry is facing a century-old conundrum from Huanglongbing (HLB) (Clark et al., 2018). Albrecht et al. (2012) evaluated the influence of 15 different rootstock varieties on HLB disease development in Florida field-grown Valencia and Early Gold sweet orange [*C. sinensis* (L.) Osb.], and they found that the highest fruit production, under high HLB pressure, were obtained from the combinations of citrus scion cultivars on US-802 and CC rootstock.

In a citrus rootstock breeding program of our team, an unexpected tetraploid CC genotype was found via seedling screening in our previous study. Tetraploid CC plants exhibit many characteristics that are superior to those of diploid plants with respect to diverse performance, and this is probably due to chromosome doubling (Oustric et al., 2019). Tetraploid CC rootstock improved the chilling stress tolerance of common Clementine mandarin (*C. clementina* Hort. ex Tan.) scion when compared to the graft of Clementine onto diploid CC rootstock (Oustric et al., 2017). Tetraploid CC trees also present more tolerance toward salinization, hence conferring greater salt tolerance to citrus scion cultivars (Ruiz et al., 2016b). Tetraploidy is associated with an increased boron-excess tolerance in CC rootstock (Ruiz et al., 2016a). In addition, tetraploid CC rootstock is more tolerant to water deficit than the corresponding diploid one (Oliveira et al., 2017). Given the importance of diploid CC plant in citrus production system and various tetraploid superiority, a comprehensive investigation into them is critically needed and of particular interest.

Each species has a characteristic chromosome complement, the karyotype (Jiang, 2019), which represents the highest level of structural and functional organization of genome (Weiss-Schneeweiss and Schneeweiss, 2013; Liang and Chen, 2015). Karyotypic features have greatly facilitated taxonomic and systematic studies, and they have also provided important insight into genome size estimations as well as genome structure and organization at the chromosomal level in numerous plant species (Chèvre et al., 2018), including radish (*Raphanus sativus* L.) (He et al., 2015), *Coix lacryma-jobi* L. and *Coix aquatica* Roxb. (Cai et al., 2014), peanut (*Arachis hypogaea*) (Zhang et al., 2016), sweet potato (Sun et al., 2019), Oleaceae plants (Luo and Liu, 2019), Clementine mandarin (Deng et al., 2019b), and blood orange [*C*. *sinensis* (L.) Osb.] (Deng et al., 2019a). Elucidating chromosome constitution and the karyotype including the number, absolute and relative size, symmetry, and centromere position of the chromosome complement in nucleus cell of individual eukaryotic species, is a fundamental question in plant biology (Liang and Chen, 2015; Jiang, 2019). Nevertheless, up to now, the chromosomal characterization of CC rootstock remains unexplored, partly because of its inherent small size and morphological similarity of mitotic chromosomes as well as the paucity of accessible chromosome landmarks in the Aurantioideae subfamily (Krug, 1943; Silva et al., 2015).

Fluorescence *in situ* hybridization (FISH) is one of the most powerful and widely used modern molecular cytogenetic techniques with which to perform chromosomal identification and karyotype analysis (Jiang, 2019). The utility of FISH depends largely on the availability of probes that allow the identification of chromosome-specific regions (Jiang, 2019; Sun et al., 2019). Repetitive DNA sequences, including tandem repeats (also known as satellite repeats), 45S ribosomal DNA (rDNA), 5S rDNA, and some oligonucleotides (Garrido-Ramos, 2017), are valuable cytogenetic markers that have been extensively developed as representative FISH probes for chromosome identification in diverse plant species (Cai et al., 2014; He et al., 2015; Jiang, 2019; Sun et al., 2019).

Several excellent reports have emphasized the important role of rDNAs, and some specific genomic fragments, such as bacterial artificial chromosomes (BACs) and rDNAs, have proved particularly useful in identifying chromosomes in some species of *Citrus* and closely related genera (Brasileiro-Vidal et al., 2007; Moraes et al., 2008; Marques et al., 2011; Mendes et al., 2011; Silva et al., 2013, 2015). As sequencing technology and associated bioinformatics tools have advanced, citrus genomics have followed suit. Recently, we analyzed the repeat sequence composition in the Clementine mandarin genome and successfully developed a set of FISH probes, encompassing a centromeric-like repeat (CL17), four major satellite repeats (CL1, CL2, CL3, and CL4), two rDNAs (45S and 5S rDNA), and a telomeric repeat (TTTAGGG)_*n*_ (Deng et al., 2019b). In that study, the physical distribution of these repetitive DNAs on the same somatic metaphase chromosomes of Clementine mandarin and other several citrus cultivars were also analyzed (Deng et al., 2019b). Subsequently, a detailed karyotype of diploid blood orange was established by multicolor FISH with these excellent probes, and we did the same for its spontaneously occurring tetraploid plant (Deng et al., 2019a).

The present study is a continuation of our recent two papers (Deng et al., 2019a, b). This study aimed to test the feasibility of repetitive DNA-based FISH on chromosome discrimination of citrus rootstock cultivars to establish the integrated somatic metaphase chromosome karyotypes of diploid and tetraploid CC rootstocks and to analyze the chromosome complement of the tetraploid CC plant. Herein, this follow-up study applied sequential multicolor FISH with the aforementioned repetitive DNAs and proposed the FISH-based karyotypes of diploid CC rootstock and its spontaneously occurring tetraploid. The results presented extend the limited knowledge of molecular cytological characterization of this extensively used citrus rootstock. Moreover, the present study also provides the fundamental basis for future exploration of genome organization of CC rootstock and intricate polyploid formation.

## Materials and Methods

### Plant Materials

The tetraploid CC plant used in this study was originally obtained from a seedling screening in the annual laboratory activity of our team. The diploid and tetraploid plants were transplanted into the field simultaneously and maintained under identical natural field conditions at the experimental base of the College of Horticulture and Landscape Architecture, Southwest University, Chongqing, China (latitude 23.39°N, longitude 34.95°E). The ploidy status of the plant materials was verified by flow cytometry in combination with somatic chromosome counting before the seedlings were transplanted.

### Somatic Chromosome Preparation

Somatic chromosome preparation was made following our previously published protocol with minor optimizations (Deng et al., 2019a). Briefly, at least 20 fresh and fine roots (approximately 3–4 cm proximal to the root tip) were carefully dissected from plants with a tweezer on a sunny morning. These excised root tips were rinsed in tap water and pretreated in ice-cold water for 24 h in dark, and this was followed by fixation in a freshly prepared 3:1 Carnoy’s fixative solution of absolute ethanol (EtOH) to glacial acetic acid for at least 24 h at room temperature (RT). The treated samples were then stored in 70% EtOH at −20°C until further use. The fixed root tips were thoroughly washed with distilled water and citrate buffer (0.01 M citric acid and 0.01 M sodium citrate buffer, pH 4.8). Thereafter, actively growing root apexes (approximately 1.5–2.5 mm long) with meristematic cells were transferred into an enzyme solution consisting of 3% (w/v) cellulose Onozuka R-10 (Yakult Pharmaceutical Industry Co., Ltd., Tokyo, Japan) plus 0.3% (w/v) pectolyase Y-23 (Kikkoman Corp., Tokyo, Japan) and digested for 1.5–2.0 h at 37°C. After enzyme treatment, the softened material was carefully rinsed in distilled water to remove excess enzymes and transferred into a drop of freshly prepared 3:1 Carnoy’s fixative solution. Each root was carefully transferred to a microscope slide and immediately squashed using a tweezer. Finally, a small drop of fixative was added to the slide and flame dried. The chromosomes of mitotic metaphase cells were examined under phase contrast microscope, and good-quality chromosomes at metaphase stage were selected for further sequential multicolor FISH experiments.

### DNA Probe Labeling and Sequential Multicolor FISH

The following seven probes, encompassing 45S rDNA, 5S rDNA, four satellite repeats (CL1, CL2, CL3, and CL4), and a centromere-like repeat (CL17), were used for sequential multicolor FISH. These seven probes, representing repetitive chromosomal markers, were previously amplified from genomic DNA of Clementine mandarin by PCR using primer pairs as detailed by Deng et al. (2019b). A digoxigenin-labeled oligonucleotide of (TTTAGGG)_3_ (Nelson et al., 2014) was directly synthesized to map telomeric sequences. Details of DNA probe labeling via nick translation and sequential multicolor FISH targeting all repetitive elements followed our reported protocol without modification (Deng et al., 2019a). Three rounds of sequential FISH experiments were performed on the same well-spread mitotic metaphase chromosomes of diploid and tetraploid CC plants. Probes (2 μL) were equally mixed before hybridization and added to the hybridization mixture, including 10 μL deionized formamide, 4 μL 50% dextran sulfate, 2 μL 20 × SSC, and 2 μL sheared salmon sperm blocking DNA (10 mg/mL; Sigma-Aldrich, St. Louis, MO, United States). A total volume of 20 μL hybridization mixture was added to each slide, and the slides were covered with coverslips of appropriate sizes. Probes and chromosomes were denatured together on a hot plate at 85°C for 1.5 min and incubated in a 37°C moist chamber at least 6 h or overnight. The procedure of FISH signal detection and posthybridization washes described by Deng et al. (2019a) without modifications were used. The slides were mounted with Vectashield^§^ antifade mounting medium containing 1.5 μg/mL 4′,6-diamidino-2-phenylindole (DAPI) (Vector Laboratories, Inc., Burlingame, CA, United States).

### Microscopy and Image Processing

The fluorescence signals were visualized using an Olympus epifluorescence microscope (BX61; Tokyo, Japan) at the same magnification times, and the photomicrographs of FISH chromosomes were captured using a Sensys CCD camera (Qimaging RetigaTM SRV Fast 1394, Vancouver, BC, Canada) under the same exposure intensity and time. Images were preudocolored (blue for DAPI, red for anti-digoxigenin-rhodamin, green for anti-avidin antibody conjugated with FITC, yellow for diethylaminocoumarin-5-dUTP), inverted and merged using ImageJ software (National Institutes of Health, Wayne Rasband, MD, United States), and further cropped and processed with Adobe Photoshop CS6.0 software (Adobe Systems Incorporated, San Jose, CA, United States) using only functions affecting the entire image simultaneously and equally.

### Statistical Analysis of Karyomorphological Indices

The chromosome number was estimated based on at least 30 split phases with well-separated and good-quality metaphase spreads. All the chromosome indices and karyotype parameters were calculated based on the mean values of at least five better split phase through KaryoType software (Altinordu et al., 2016). A total of 13 independent karyotype parameters, including long arm length, short arm length, arm ratio, relative length of chromosome length, and centromeric index, were calculated for both diploid and tetraploid CC plants. Chromosome classifications were determined based on definitions provided by Levan et al. (1964). A karyotype idiogram was produced based on the decreasing relative chromosome lengths and centromere positions, or a combination of these two aspects of chromosome morphology, as well as the position and distribution of other chromosomal landmarks, in reference to previous reports of Cai et al. (2014), He et al. (2015), and (Deng et al., 2019a, b).

## Results

### Physical Localization of Highly Repetitive DNA Sequences on Somatic Chromosomes of Carrizo Citrange

**FIGURE 1:**
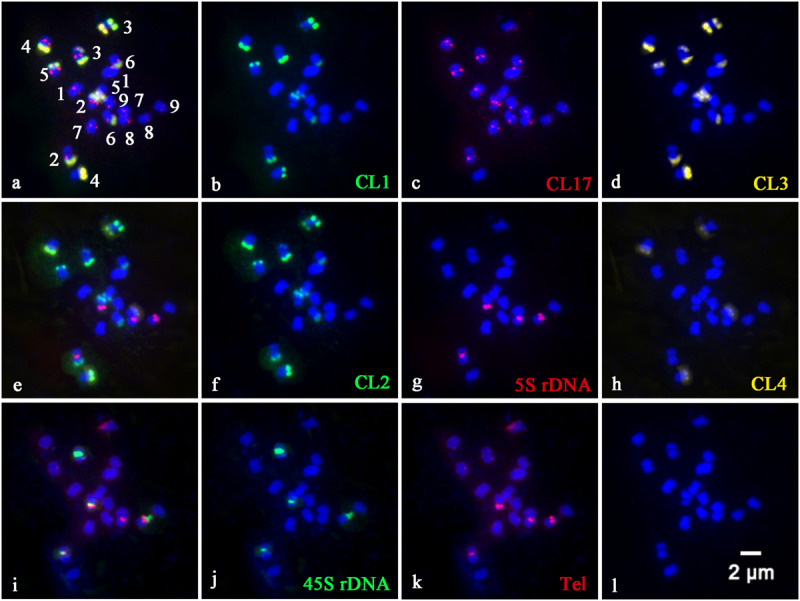
Physical localization of different highly repetitive DNA sequences on root tip metaphase chromosomes of diploid Carrizo Citrange rootstock. The merged images of **(a)**, **(e)**, and **(i)** came from the hybridization sites in **(b–d)**, **(f–h)**, and **(j,k)**, respectively. **(b)** Satellite repeat CL1 (green), **(c)** a centromere-like repeat CL17 (red), **(d)** satellite repeat CL3 (yellow), **(f)** satellite repeat CL2 (green), **(g)** 5S rDNA (red), **(h)** satellite repeat CL4 (yellow), **(j)** 45S rDNA (green), and **(k)** telomere repeat (red) were used as FISH probes. Signal color corresponds to probe name in **(b–d,f–h,j,k)**. **(l)** DAPI-stained chromosomes were converted into a blue image. Bar = 2 μm.

**FIGURE 2:**
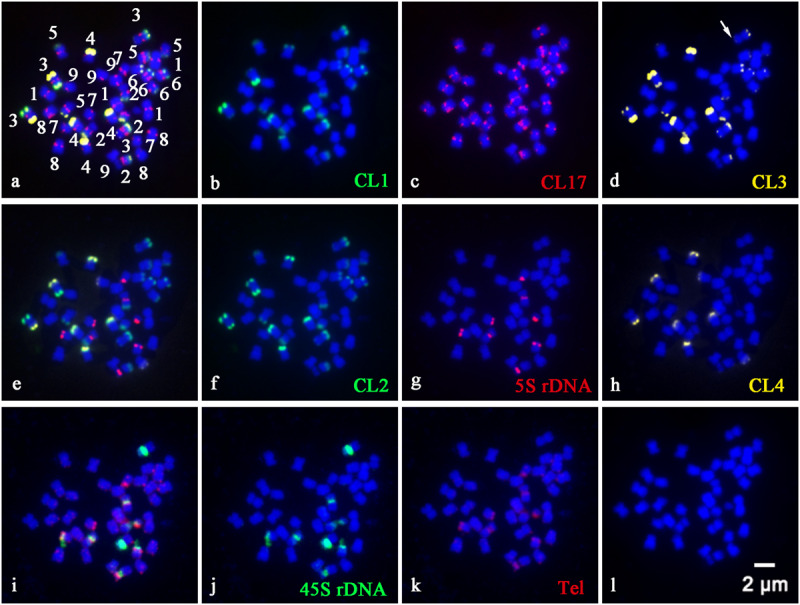
Physical localization of different highly repetitive DNA sequences on root tip metaphase chromosomes of tetraploid Carrizo Citrange rootstock. The merged images of **(a)**, **(e)**, and **(i)** came from the hybridization sites in **(b–d)**, **(f–h)**, and **(j,k)**, respectively. **(b)** Satellite repeat CL1 (green), **(c)** a centromere-like repeat CL17 (red), **(d)** satellite repeat CL3 (yellow), **(f)** satellite repeat CL2 (green), **(g)** 5S rDNA (red), **(h)** satellite repeat CL4 (yellow), **(j)** 45S rDNA (green), and **(k)** telomere repeat (red) were used as FISH probes. Signal color corresponds to probe name in **(b–d,f–h,j,k)**. **(l)** DAPI-stained chromosomes were converted into a blue image. Bar = 2 μm.

In order to precisely identify each pair of homologous chromosomes and obtain detailed karyotypes of diploid and tetraploid CC plants, the physical distribution of highly repetitive DNAs on somatic chromosomes was determined by sequential multicolor FISH on the mitotic metaphase chromosomes coming from the same metaphase spreads (Figures 1, 2). Some other additional metaphase plates produced consistent results with respect to chromosome counts, and both signal numbers and locations are used for karyotype analysis, as shown in Supplementary Table S1. Somatic chromosome images at mitotic metaphase stages of the analyzed diploid and tetraploid CC plants are shown in Figures 1, 2, respectively. In general, the different probes distributed to various positions and showed great variation in the numbers and fluorescence intensities of hybridization sites (Figures 1, 2). FISH signals of highly repetitive DNAs in the tetraploid CC plants (Figure 2) corresponded quantitatively to the double signals of its metaphase counterpart in the diploid CC rootstock (Figure 1).

CL1 (Figure 1B), CL2 (Figure 1F), CL3 (Figure 1D), and CL4 (Figure 1H) signals were located predominantly in subtelomeric regions of several homologous chromosome pairs, with 11, 12, 11, and 4 hybridization sites being observed in diploid CC rootstock, respectively (Figure 1). Specifically, FISH with a CL1 probe revealed an asymmetrical pattern that the CL1 loci were localized in the subtelomeric regions of the long arms of the chromosome pairs 2, 3, 4, 5, and 6, and only one short arm of the chromosome pair 3 (Figures 1B, 2B). Positive signals of CL2 were visible at the subtelomeric regions of the long arms of the chromosome pairs 2, 3, 4, 5, and 6, and the two short arms of chromosome pair 3 (Figures 1F, 2F). CL3 FISH signals (Figures 1D, 2D) were co-localized with CL2 locus. CL4 FISH signals were detected in the subtelomeric regions of two long arms of chromosome pair 4, only one short arm of the chromosome pair 3 co-localized with other three satellite repeats, and only one long arm of the chromosome pair 7 (Figures 1H, 2H).

The CL17 probe hybridized to sites on chromosome centromeric regions (Figures 1C, 2C). FISH with the oligonucleotides of (TTTAGGG)_3_ showed noticeable twin fluorescent signals at the terminal regions of all chromosomes in both diploid (Figure 1K) and tetraploid CC rootstock (Figure 2K). A few weak signals of the oligonucleotides of (TTTAGGG)_3_ were also detected in interstitial regions of several chromosome pairs, but there were much less intense than those in the terminal regions (Figures 1K, 2K). FISH with 45S rDNA showed four hybridization sites in diploid CC rootstock, with two sites on the short arms of chromosome pair 2 corresponding to one pair of nucleolus organizer regions (NORs), one site on one short arm of chromosome pair 3 co-localized with other four satellite repeats, and one site occurred on the chromosomal satellite position next to the short arm of chromosome pair 8 (Figure 1J). Two 5S rDNA sites were identified in the pericentromeric regions of the short arms of chromosome pair 2 co-carrying 45S rDNA locus, and another two 5S rDNA FISH signals were found in the pericentromeric regions of the short arms of chromosome pair 8 (Figure 1G). The tetraploid CC rootstock had the same rDNA distribution pattern but twice as many loci as the diploid CC rootstock (Figures 2J, 2G). Chromosomes stained by DAPI are shown in Figures 1L, 2L, from which we can see it is hard to recognize the chromosome pair for the small size and high similarity in morphology without the aids of chromosome landmarks.

### Characterizing Karyotype of Diploid and Tetraploid Carrizo Citrange Chromosomes

**FIGURE 3:**
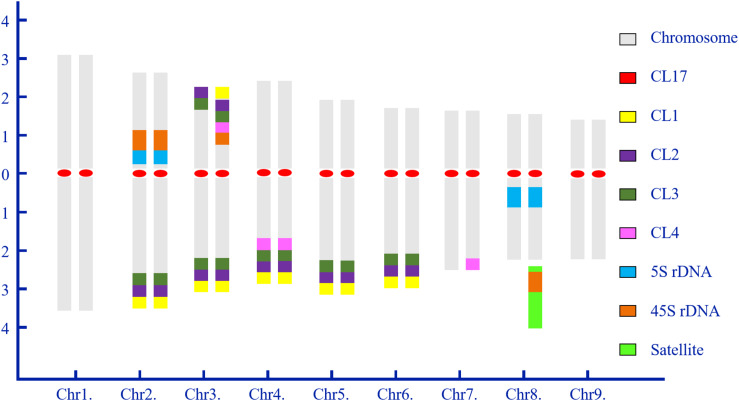
Idiogram of diploid Carrizo Citrange rootstock showing the chromosome complement and relative positions of highly repetitive DNA sequences. Numbers on the *x*- and *y*-axis indicate the numbers of homologous chromosome pairs and the relative arm length of chromosome pairs, respectively. The ideogram is construed based on the sequential multicolor FISH-based karyotype. Chromosomes are arranged in matched, homologous pairs and ordered by the entire length from the longest to shortest and centromere positions, and a combination of these two aspects of chromosome morphology. The color scheme at right side shows the color of each probe as represented in this idiogram.

Chromosome numbers were confirmed by chromosome counts in at least 30 mitotic metaphases. All of the examined mitotic metaphase plates of CC rootstock showed a fundamental number *x* = 9 (Figures 1, 2). Karyotypes of CC individuals possessed the same standard diploid constitution 2n = 18 (Figure 1) and tetraploid constitution 4n = 36 (Figure 2). The karyotype parameters, chromosome classification, and morphological features were detailed in Supplementary Table S1. The karyotype formula of CC rootstock was 2n = 2x = 12m (1sat) + 6sm. The chromosome complement consisted of three pairs of submetacentric chromosomes and six pairs of metacentric chromosomes, with one chromosome being satellite chromosome. Concerning Stebbin’s classification of karyotypes, CC rootstock falls into the 2B category (Supplementary Table S1). The homologous chromosomes were paired based on chromosome size and morphology, centromere position, and the presence and distribution of repetitive DNA loci, as shown in Figures 1, 2, collectively facilitating the integration of karyotype of CC rootstock (Figure 3 and Supplementary Table S1).

From Figure 3, the karyotype results convincingly showed that diploid CC rootstock is a hybrid. In the karyotype, chromosomes were ordered by a gradual decrease excluding the satellite length in computing arm ratios. Thus, chromosome pairs 1 and 9 can be recognized by the largest and smallest size among all the homologous chromosome pairs, respectively. It is evident that chromosome pair 2 carried a pair of strong 45S rDNA loci co-located with 5S rDNA at the NOR of short arms and three pairs of satellite DNA repeat (CL1, CL2, and CL3) loci at the terminal regions of long arms. Chromosome pair 3 was characterized by a pair of robust CL2 and CL3 loci on the terminal positions of both short and long arms; one 45S rDNA locus, one CL1 locus, and one CL4 locus were terminally positioned in only one short arm of chromosome pair 3 near the CL2 and CL3 loci. Chromosome pair 4 could be easily distinguished by four pairs of satellite repeat loci on the long arms. Morphologically, chromosome pairs 5 and 6 resembled each other and presented symmetrical CL1, CL2, and CL3 signals at the subtelomeric regions of long arms. Only one long arm of chromosome pair 7 contained a CL4 locus. Chromosome 8 showed strong 5S rDNA signals in the pericentromeric regions of long arms, with one satellite chromosome being labeling by intense 45S rDNA signal next to the long arm. The corresponding tetraploid CC chromosome pairs could also be identified for these chromosomal landmarks, and, therefore, only the idiogram of diploid CC is presented.

## Discussion

The small size (between 2 and 4 μm) and similar morphology of *Citrus* chromosomes (Krug, 1943) has been long challenging unequivocal identification of homologous chromosome pairs (Silva et al., 2015). Krug (1943) investigated the first chromosome characterization of *Citrus*. Since then, chromosome banding techniques, such as C-banding (Liang, 1988) and CMA^+^/DAPE^–^ fluorescence banding (Carvalho et al., 2005; Brasileiro-Vidal et al., 2007; Guerra and Souza, 2009; Silva et al., 2010; Marques et al., 2011; Mendes et al., 2011), and the combination of CMA banding and rDNA- and BAC-based FISH (Carvalho et al., 2005; Brasileiro-Vidal et al., 2007; de Moraes et al., 2007; Guerra and Souza, 2009; Silva et al., 2010, 2013; Marques et al., 2011; Mendes et al., 2011) have gradually been developed for citrus cytogenetic study over time.

Taking advantage of the availability of citrus reference genome sequences and using the newly developed bioinformatic tools, we have investigated the content and the types as well as the proportional contribution of repetitive DNAs in *C. clementina* mandarin genome (Deng et al., 2019b). The chromosome distribution patterns of these highly repetitive DNAs on mitotic chromosomes of *C. clementina* mandarin and several citrus scion cultivars within *Citrus, Poncirus*, and *Fortunella* were analyzed through FISH (Deng et al., 2019a, b). In the present study, we extended the FISH-based karyotype analysis to one of the most widely used citrus rootstocks and its spontaneously occurring tetraploid plant. While the previous karyotype studies focused on citrus scion variety, the karyotype of citrus rootstock and its corresponding tetraploid plants were studied for the first time in the present study.

FISH technique has been successfully applied to identify specific chromosome pair or chromosome regions in a number of plant species (Cai et al., 2014; He et al., 2015; Zhang et al., 2016; Deng et al., 2019a, b; Jiang, 2019; Luo and Liu, 2019; Sun et al., 2019). The results in the present study confirmed the importance and advantage of using repetitive DNA-based FISH for chromosome discrimination and cytogenetic characterization in CC rootstock. Accordingly, the chromosome complement of CC rootstock was clearly unraveled (Figures 1–3). The chromosome numbers (2n = 18, Supplementary Table S1) were in accordance with previously published citrus diploid data (Krug, 1943; Silva et al., 2010; Marques et al., 2011).

The repetitive DNA probes developed in our previous work (Deng et al., 2019b) were of great assistance in investigating the cytological characterization of diverse citrus scion cultivars (Deng et al., 2019a). Here, when this set of eight such probes was applied to a sequential multicolor FISH-based karyotype of CC rootstock, and the results collaborated that eight such repetitive DNAs are good chromosomal landmarks for citrus rootstock species (Figures 1–3). In this cytogenetic methodology, it is able to simultaneously reveal the diverse features of the small or difficult-to-distinguish chromosomes of citrus rootstock. Repetitive DNAs are responsible for the main components of the structural and functional organization of higher plant genomes, resulting in the genome size variation (Guerra et al., 2000; Garrido-Ramos, 2017). The tandemly repetitive DNA sequences exhibit characteristic chromosomal locations, usually at pericentromeric, centromeric, and subtelomeric regions of eukaryotic chromosomes (Cai et al., 2014; He et al., 2015). CL17 loci are valuable chromosome centromere markers for CC rootstock, and the physical mapping of CL17 loci using *in situ* hybridization allows for the determination of centromere position of chromosome pairs in CC rootstock (Figures 1C, 2C).

The repetitive DNAs could be specific for a family or genus taxonomically or may be specific for a plant species, genome or even a chromosome pair (Jiang, 2019). Thus, the distribution of repetitive DNAs may vary qualitatively and quantitatively between citrus taxa (Guerra et al., 2000). The genome changes may be intriguingly associated with elimination and amplification of some types of repetitive DNAs, such as tandemly arranged DNA sequences (rDNA and satellite repeats) or dispersed (DNA transposons and retrotransposons) (Garrido-Ramos, 2017). In this study, satellite repeats (CL1, CL2, CL3, and CL4) showed subtelomeric preferences in the root tip metaphase chromosome material of both diploid (Figures 1B,D,F,H) and tetraploid (Figures 2B,D,F,H) CC rootstock, which has been considered as a common and typical feature of satellite repeat in *Citrus* L (Silva et al., 2010; Marques et al., 2011; Deng et al., 2019a, b).

Subtelomere, the regions proximal to telomeres, of most eukaryotes harbor fast-evolving gene family usually in relation to adaptive processes (Brown et al., 2010). Specifically speaking, the subtelomere of common bean (*Phaseolus vulgaris*) favors the rapid evolution of genes associated with resistance (Chen et al., 2018). Satellite repeat represents a fast-evolving portion of various plant species (Cai et al., 2014; He et al., 2015). Therefore, it must be extremely interesting to understand what the possible biological functions behind this preferential subtelomere localizations of satellite repeats in citrus in the near future and how the satellites have undergone rapid evolution within *Citrus* L. genus.

Another major tandem repetitive DNA in CC genome is rDNA (Figures 1G,J, 2G,J). Results of the cytogenetic studies have been consistent in previous views that rDNA is a very useful marker to identify chromosomes and species (Silva et al., 2010; Marques et al., 2011; Deng et al., 2019a, b), as rDNAs are presented in numerous repeated units, generally organized in clusters and are thus easily visualized in plant chromosomes (Garrido-Ramos, 2017). In our study, the rDNA hybridization signals presented in only one of the homologous chromosomes (Figures 1G,J, 2G,J) explicitly supported the generic hybrid nature of CC rootstock (Deng, 2008) from cytological aspect. The 45S rDNA, generally confined to NOR (Marques et al., 2011; Lan et al., 2016), was consistently located proximally on the short arms of chromosome pair 2 in our study (Figures 1J, 2J), which may have potential to illustrate several issues of chromosomal structure and composition. The plant-type telomere probe (TTTAGGG)_3_ (Nelson et al., 2014) efficiently hybridized to the ends of all chromosomes (Figures 1K, 2K), indicating the presence of TTTAGGG repeat in CC telomere.

Chromosome doubling may generate during some period of seed formation in *Citrus* L., and tetraploid seedling may arise spontaneously in the nucellar cells. These clones are regarded to be genetically identical because they have the same genome expression profile (Cameron and Soost, 1970; Deng et al., 2019a). The distribution pattern of repetitive DNAs in the tetraploid metaphase chromosomes (Figure 2) is concordant with those in the diploid CC plant (Figure 1). The number of repetitive DNAs sites strictly correlated with the ploidy, and the tetraploid (Figure 2) possessed twice the number of sites as the diploid (Figure 1). Previous molecular study using SSR markers has been applied to hundreds of spontaneous tetraploid citrus lines, and showed that in the absence of tetraploid pollinator spontaneous tetraploid lines resulted from somatic chromosome doubling and that the rates were affected by both genotype and the environment (Aleza et al., 2011). These results presented herein confirmed that the naturally occurring tetraploid CC material arose from somatic chromosome doubling from the cytogenetic aspect. In apomictic citrus germplasm, tetraploidization is a common event, and the autotetraploids arising from chromosome doubling are extensively used as male parents in citrus improvement (Aleza et al., 2011). Further molecular cytogenetic studies, including the parental species of CC rootstock and comparative studies regarding the genome sequencing, are highly needed to elucidate a more accurate genome structure of this genetic hybrid. This results in the present study have provided valuable cytogenetic information for CC rootstock, which could be a good starting point for future chromosome engineering-based citrus rootstock improvement.

## Conclusion

In conclusion, sequential multicolor FISH with three sets of efficient probes containing a centromere-like repeat, four satellite repeats, two rDNAs, and an oligonucleotide of telomeric (TTTAGGG)_3_ repeat were conducted on the same root tip metaphase chromosomes, by which each chromosome pair was unequivocally identified. The asymmetric distribution patterns of the repetitive DNA sequences demonstrated the hybrid origin of CC rootstock. This study revealed, for the first time, the integrated karyotype and chromosomal characteristics of one most widely used citrus rootstock, Carrizo citrange (*Citrus sinensis* L. Osbeck × *Poncirus trifoliata* L. Raf.), as well as its spontaneously occurring tetraploid plant.

## Data Availability Statement

All datasets generated for this study are included in the article/Supplementary Material.

## Author Contributions

HD, GL, and XL conceived the research, reviewed and edited the manuscript, and acquired funding. HD, LL, DL, HX, QD, JW, and ZC performed the methodology optimization and data curation. HD, GT, NX, and ZG conducted the experiment and managed the plant material collection. HD wrote the manuscript. All authors read and approved the final manuscript.

## Conflict of Interest

The authors declare that the research was conducted in the absence of any commercial or financial relationships that could be construed as a potential conflict of interest.
